# Annotation of Functions of Sequences of Concern and Its Relevance to the New Biosecurity Regulatory Framework in the United States

**DOI:** 10.1089/apb.2023.0030

**Published:** 2024-09-18

**Authors:** Gene D. Godbold, Matthew B. Scholz

**Affiliations:** ^1^ Signature Science, LLC, Charlottesville, Virginia, USA.; ^2^ Signature Science, LLC, Austin, Texas, USA.

**Keywords:** controlled vocabulary, microbial pathogenesis, ontology, synthetic DNA screening, dual-use research of concern

## Abstract

**Introduction:**

Recent regulations from United States Government agencies reshape the screening of synthetic nucleic acids. These take a step away from categorizing hazard on the basis of “bad” taxa and invoke the function of the sequence in pathogenesis or intoxication. Ascertaining functions related to pathogenesis and distinguishing these from other molecular abilities that are unproblematic is not simple. Some have suggested that this information can be readily obtained from existing databases of pathogens.

**Objectives:**

We evaluate how virulence factors are described in current databases of pathogens and their adequacy for biothreat data science. We discuss limitations of how virulence factors have been conceived and propose using the sequence of concern (SoC) term to distinguish sequences with biothreat from those without. We discuss ways in which databases of SoCs might be implemented for research and regulatory purposes. We describe ongoing work improving functional descriptions of SoCs.

**Methods:**

We assess the adequacy of descriptions of virulence factors in pathogen databases following extensive engagement with the literature in microbial pathogenesis.

**Results/Conclusions:**

Descriptions of virulence factors in pathogen databases are inadequate for understanding biothreats. Many are not biothreats and would not be concerning if transferred to another pathogen. New gene ontology terms have been authored, and those specific to pathogenic viral processes are being generalized to make them relevant to other pathogenic taxa. This allows better understanding by humans and better recognition by machines. A database of annotated functions of SoCs could benefit the evolving biosecurity regulatory framework in the United States.

## Introduction

New guidance issued on October 13, 2023 from the Department of Health and Human Services (DHHS) on screening synthetic nucleic acids indicates that a sequence of concern (SoC) is one that is “…known to contribute to pathogenicity or toxicity even when not derived from…[a] regulated biological agent.”^1^ The new guidance from DHHS represents a shift in regulation from an approach based solely on taxonomy to one based on sequences from any microbe “…that may contribute to pathogenicity or harm if introduced into new genetic frameworks.”^1^ The necessity for this move has been recognized by different groups.^2,3^ A recently issued (October 30, 2023) executive order calls for the development of best practices to “manage sequence-of-concern databases to support…screening.”^4^

Infections are caused by pathogenic organisms and microbes. Pathogens are distinguished from their nonpathogenic relatives, including nonpathogenic strains of the same species, by specific molecules (carbohydrates, lipids, proteins, and combinations thereof, as well as small RNAs) that provide them with the ability to exploit particular hosts.^5,6^ These sequences, often called virulence factors, play essential roles in infectious diseases.^7^ In the past, researchers have labeled genes necessary for a pathogen to maintain its viability in a host as a “virulence factor,” leading to the description of metabolic and structural genes as virulence factors when their deletion resulted in a “loss of virulence.” This has included genes, such as those that encode bacterial siderophores, that enable the pathogen to survive in the host environment, although they do not deleteriously affect the host. Many sequences called “virulence factors” are found in both pathogens and nonpathogens, and the misleading terminology can confuse assessments of which sequences are hazardous for hosts and important in pathogenesis.^8,9^ Some have proposed that virulence sequences be divided into “true virulence” factors, “virulence-associated” factors, and “virulence life-style” factors.^7^

Following a 2010 NRC report,^10^ we employ the SoC term to designate encoded molecules that could be hazardous biothreats—contributing to pathogenicity or harm—if transferred to another microbe. For reasons we discuss in greater detail below, we believe that SoC is more useful as a designator of hazardous biothreat function than virulence factor, although there is considerable overlap.

Our group was one among several who worked on the computational side of the functional genomic and computational assessment of threats (Fun GCAT) program.^11^ From 2017 to 2022, the program funded tool development to answer the following three questions about any given sequence: (1) From what taxon did it originate? (2) What were its biological functions? and (3) How dangerous was it? We realized that, for pathogen sequences, the danger inherent in a sequence was dependent on what antihost functions it possessed in the pathogen. If a sequence had a concerning function in one organism, then it would probably maintain that function when moved to another organism. We realized that “dangerous” sequences were found primarily among virulence factors from pathogenic microbes and secondarily from venom-producing taxa.

As we embarked on the task of discerning “dangerous” sequence functions, we discovered that, for nonviral and nontoxic sequences, there was no set of terms that could be systematically employed to distinguish concerning from innocuous sequences.^12^ Some of this was the result of the ways researchers applied the term “virulence factor.” We also found that a majority of sequences with concerning functions reported in the literature were not annotated for those functions in public databases, even when the sequences themselves were present.

In this article we discuss annotations of virulence factors in sequence databases. By annotation, we mean information on the function of a sequence that are the result of published investigations and that are associated with the sequence in some type of dataset. The annotation can be formalized in either a controlled vocabulary or free text. We address how we approached annotating sequences with functions that are dangerous—which we denote as SoCs. We describe our ongoing attempts to improve the vocabularies describing these functions and what we think remains to be done. We also suggest benefits that a more granular annotation of the concerning functions of SoCs (FunSoCs) will provide for a biosecurity regulatory framework.

## Pathogen Databases and Virulence Factor Annotation

Because of their outsized historical impact on human populations, microbial pathogens are of considerable interest. There are some longstanding databases devoted to exploring microbial pathogens, including the bacterial and viral bioinformatics resource center (BV-BRC)^13^ and Eukaryotic Pathogen, Vector and Host Informatics Resource (VEuPathDB).^14^ These house pathogen genomes and appropriate bioinformatic tools. While these are excellent supports for the generic study of pathogens, they do not specialize in annotation for virulence factors.

### Bacterial and Viral Bioinformatics Resource Center

The BV-BRC is one of the bioinformatic resource centers (BRCs) supported since 2004 by the National Institute of Allergy and Infectious Disease (NIAID) of the U.S. National Institutes of Health (NIH).^13^ The BV-BRC was recently formed from a merger of the pathosystems resource integration center (PATRIC), the bacterial BRC, with the influenza research database (IRD) and the virus pathogen resource (ViPR), the viral BRCs.^15^ The BV-BRC has over 140,000 sequences with the property of “Virulence Factor” drawn from Victors (discussed below), nearly 80,000 sequences from “PATRIC_VF,” and nearly 42,000 sequences taken from the Virulence Factor Database (VFDB) (discussed in VFDB). The function of the virulence factors housed in the BV-BRC is often difficult to discern from the information provided. The information categories of “Product” and “Function” in the BV-BRC database are the most helpful for understanding the potential function of a protein. But these data are not focused on explicating the role of the sequence as a virulence factor; the “Function” category is usually vacant. BV-BRC uses the Rapid Annotation using Subsystem Technology (RAST) Toolkit to annotate their bacterial genomes^16^ and Viral Genome ORF Reader (VIGOR4) to predict protein sequences for 10 viral genus or species. The original GenBank annotations are propagated for other viruses, and these are not particularly concerned with the role in virulence.

### Eukaryotic Pathogen, Vector and Host Informatics Resource

Like the BV-BRC, VEuPathDB, is a large bioinformatic resource center that has resulted from several mergers.^14^ Eukaryotic pathogens are extraordinarily diverse, and VEuPathDB does a wonderful job of grappling with the profusion of species. Unlike other pathogen databases it also contains datasets of particular host and vector species.^17,18^ Although the resource contains annotations from the gene ontology (GO), it does not supply additional data or analysis on the pathogenic function of individual genes from the datasets from which it pulls information. There does not appear to be as much basic research investigating virulence factors from eukaryotic pathogens for humans compared with bacterial and viral SoCs. This rough assessment is based on annotating just 100 SoCs from eukaryotic pathogens of humans compared with the thousands we have found from bacteria and many hundreds from viruses.

## Virulence Factor Annotation: PHI-Base, VFDB, and Victors

These resources are much more modest in scope than the BV-BRC or VEuPathDB, but they have exerted themselves to supply or present extra information on virulence factors in a way the larger databases have not. BV-BRC uses the VFDB and Victors data to annotate its sequences.

### Pathogen–Host Interaction Database

The Pathogen–Host Interaction database (PHI-base) has a rich set of manual annotations from more than 4300 publications detailing the effects of mutagenesis studies of a gene or pathogen and how that affects the interactions of the encoding pathogen with a particular host. PHI-base has a slight preponderance of plant hosts (and pathogens), which covers a real need for more descriptions of plant pathogen–host interactions and differentiates it from similar resources.^19^ A typical row of the dataset contains a protein, the gene that encodes it, the strain of pathogen encoding the gene, the gene function, the host exploited by the pathogen, and the phenotype manifested when the gene is mutated (frequently a deletion). There are nine controlled phenotype descriptions: “loss of pathogenicity,”“reduced virulence,” “unaffected pathogenicity,” “increased virulence,” “effector,” “lethal,” “enhanced antagonism,” “resistant to chemical,” and “sensitive to chemical.”^20^ Although most of these would not be useful for functional assessments of biothreat, the “effector” designation signifies a *bona fide* SoC.

### VFDB

This resource was first published in 2005^21^ and there have been several updates.^22,23^ For the 2022 version of the virulence factor database, the authors improved the granularity of their virulence descriptions for the ∼22 species of bacterial pathogens of humans they maintain. These descriptions employ the following categories, with subcategories in parentheses:^24^

1. adherence (fimbrial, nonfimbrial)
2. invasion
3. effector delivery system (types II–VII)
4. motility (flagellar, intracellular)
5. exotoxin (membrane-acting, intracellularly active)
6. exoenzyme (hyaluronidase, kinase, coagulase, lipase, protease, nuclease)
7. immune modulation (antiphagocytosis, serum resistance, immunoglobulin, antigen variation, apoptosis, inflammatory signaling pathway)
8. biofilm (formation, quorum sensing)
9. nutritional/metabolic factor (metal uptake, metabolic adaptation)
10. stress survival
11. post-translational modification
12. antimicrobial activity
13. regulation

Although many of these virulence factors are involved in disrupting host homeostasis and subverting barriers and innate immunity, the sequences tagged with biofilm formation, quorum sensing, stress survival, post-translational modification, antimicrobial activity, regulation, and all the enzyme activities listed for exoenzymes are not exclusive to pathogenic organisms and are not generally pertinent to loss of homeostasis in a host.^8^ These sequences might be better assigned to a “virulence lifestyle” category instead of virulence factors properly understood.^7^

In addition, the VFDB authors appear to neglect the adjudication of virulence factors with multiple functions. The fixed color scheme they employ cannot accommodate multifunction sequences.^24^ In their recent machine learning project, they appear to have excluded sequences with multiple pathogenic functions before classifying them.^25^ This is a problem if one is attempting to garner functional information on virulence from the VFDB, as it is not clear which functions are neglected. Many SoCs have two or more disparate functions.^12,26^ Out of 1900 bacterial sequences we have annotated, 30% have at least two pathogenic functions, 13% have three, and 2.5% have five or more.

The VFDB has an “effector” subcategorization for the proteins excreted through secretion systems by bacteria. This designation, while useful and pertinent, neglects the myriad pathogenic functions of these interesting sequences, particularly in immune subversion and the hijacking of eukaryotic endomembrane systems. Another critique regards the integration of the virulence designations with their literature citations and the sequences that bear those designations. The VFDB authors abjured manual curation in their 2019 article;^23^ therefore, the functional blurbs are either taken indirectly from other, aggregated data sources or is a legacy of earlier curation efforts.

### Victors

Victors is a searchable, online database of genes with published evidence of involvement in virulence.^27^ As of this writing, it contains 5304 manually curated virulence factors. Victors uses the clusters of orthologous genes (COGs) classification for all entries in their database,^28^ although these functions are not specific to virulence. The manual annotation for a gene is provided as “Molecule Role Annotation.” This provides a one-line description of experimental evidence from the deletion of the gene for the pathogen affects pathogenesis in a host usually absent of suggested gene function. This is not particularly informative for understanding its role(s) as a biothreat.^7,12^

## The False-Positive Problem; Distinguishing Virulence Factors from Sequences of Concern; Which SoCs Are the Most Concerning?

A sequence can be critical for the survival of a pathogenic microbe in a host (i.e., siderophores, antimicrobial resistance in a patient on antibiotics) and called a virulence factor for that reason. Yet those sequences do nothing to deleteriously affect host homeostasis or otherwise contribute to pathogenesis. These types of sequences can also be found encoded in microbes that have no pathogenic capacity and so they are essentially false-positives that regularly appear in bioinformatic screens for virulence.^7–9^

Despite 50 years of research in microbial pathogenesis, we cannot calculate or predict the severity of disease that will be produced in a host organism by a particular microbe with a given set of encoded virulence factors. Very few articles have been published in which the pathogenicity of a microbe with a given set of virulence factors is compared with a nearly isogenic microbe with a slightly different set.^29^ Pathogenesis is an emergent property of the host–parasite interactions shaped by stochastic and environmental factors and the development of the host immune response.^30–32^ However, this large body of research, consisting of many thousands of articles, has shown that virulence factors are necessary for disease.^29,33^ We and others have been seeking to standardize and automate the detection of biothreat sequences from microbial pathogens in large sequence datasets through custom software.^12,34,35^ This is necessary for better synthetic DNA screening, pathogen surveillance through genomic epidemiology, and improved recognition of dual-use research of concern (DURC).^26^

## The Importance of Functional Annotation for Sequences of Concern

Our annotation pipeline and the resulting dataset has been specifically fashioned to provide critical context for understanding how a particular sequence contributes to pathogenicity and how it might be harmful if introduced into a new microbe. This assessment is typically based on the documented contribution of that sequence to pathogenicity in the original microbe, although in ∼100 cases, researchers have demonstrated the same function in another microbe.^12^ We believe this assessment can be used to judge whether a synthetic DNA sequence poses a biothreat.^35^ SoCs from pathogens contribute to the loss of host homeostasis by (1) directly damaging the host, (2) subverting host immunity, (3) disseminating within host tissues, (4) adhering to host cells or tissues, (5) invading host cells, (6) creating intracellular niches, or (7) otherwise manipulating host cell biology.^12,26^

We believe our annotations are likewise helpful for ascertaining if research with a sequence has the potential to fall under DURC oversight policies since each specifies at least one way, supported by at least one publication, in which the sequence specifically—*not* generally—contributes to host pathogenesis. The DURC policies specify that research that is either conducted or funded by a federal agency on 15 pathogens and toxins that is “reasonably anticipated” to produce one of seven experimental outcomes are subject to review by the funding agency.^36,37^ Because the range of pathogens and toxins with SoCs is not exhausted by this list of 15, we anticipate that this list could be broadened, similar to what has occurred for synthetic DNA screening, to include any sequence that may contribute to pathogenicity or harm. If such a change is instituted, then listing harmful functions for such sequences, with supporting documentation, could help determine if a sequence is “reasonably anticipated” to lead to problems in the context of alteration within the original microbe or transfer to a different one.^26^

### Sequence of Concern Annotation: Past, Present, and Future

**Figure 1.**
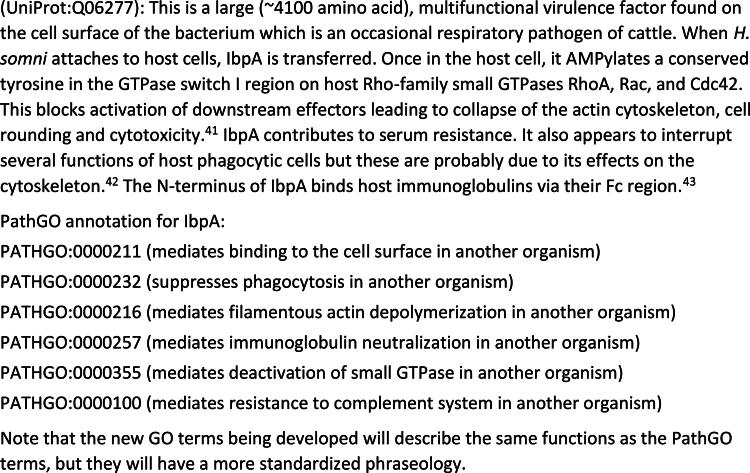
Annotation for immunoglobulin-binding protein A of *Histophilus somni*.

We have been linking UniProt accessions with one or more FunSoCs and citations from the professional literature describing the function of that sequence. These ∼30 functional tags designed for biothreat determination are supplemented with more granular terms from a controlled vocabulary, the Pathogenesis Gene Ontology, developed by researchers at the Johns Hopkins University Applied Physics Laboratory for Fun GCAT.^38^ We have annotated over 2400 microbial pathogen sequence types with ∼140 PathGO terms. An example of this annotation is given for immunoglobulin-binding protein A (IbpA), a multifunctional SoC from *Histophilus somni*, a bacterial pathogen of bovines, in Figure 1.

Although the PathGO terms were rooted in biological process and molecular function terms of the GO to clarify the host molecules and pathways that are the targets of microbial SoCs, the scope of the PathGO effort disallowed further integration with GO and foreclosed the possibility that the PathGO terms could be used in public databases (e.g., UniProt). To devise a set of terms applicable to many pathogens as well as venom components, we have begun adapting the PathGO terms to GO biological process terms, prioritizing those terms for which many examples had been published. These terms call out molecular pathways of the host that are affected by SoCs. The syntax employed is generalizable to “symbiont-mediated (perturbation/suppression/activation) of host [*biological process*]”. We intend to deposit a set of SoCs annotated with the new GO terms into UniProt in mid to late 2024. This should assist the systematization of descriptions of microbial pathogenesis so that both machines and humans can better recognize them. We hope it will lead to general improvements in SoC annotation in secondary and composite databases. We anticipate this will allow bioinformaticians, systems biologists, and other biological data scientists to investigate commonalities across a range of hosts and symbionts. Such an assessment of microbial pathogens of humans may lead to advances in countermeasures to defend host targets that are often subverted by diverse microbes (e.g., nuclear factor kappa-light-chain-enhancer of activated B cells (NF-κB) signaling, interferon signaling, antigen presentation). We are open to collaborating with other groups with similar microbial datasets to advise on how they might be best standardized.

## Benefits of a Biosecurity Regulatory Framework, Including Datasets of Sequences of Concern

The benefits of a regulatory framework based on a list of verified SoCs instead of taxonomy is that the sequences on such a list possess a published function detailing their pathogenic potential when expressed in a particular context. For example, SpyCEP from *Streptococcus pyogenes* is a nontoxic protease that degrades a critical host chemokine responsible for coordinating immune defense. SpyCEP allows *Streptococci* to disseminate better within the host. When expressed by the nonpathogenic *Lactococcus lactis*, SpyCEP renders the bacterium pathogenic in a mouse leg wound model.^33^ Because of the functional selection criterion, an approach based on SoCs helps both researchers and regulatory boards understand the answer to the “reasonably anticipated” guidance. Every sequence on the list is there because it is reasonably anticipated to make a microbe worse in a defined fashion. We have attempted to show how this might work in practice for DURC.^26^ A list of SoCs should make the job of adjudication of potential DURC and nonviral enhanced potential pandemic pathogens (ePPPs) easier and more straightforward for Biosafety Review Boards and other panels who face the nontrivial assignment of parsing research with biosecurity implications.

Another benefit to this change for DURC/P3CO oversight is that it will allow the deregulation of most sequences from microbes on the BSAT and nonviral ePPP lists. While *Bacillus anthracis* encodes ∼5800 genes, only ∼14 are directly involved in pathogenesis.^26^ Most of what is encoded within bacterial and fungal genomes are not SoCs. The vast majority are “housekeeping” genes concerned with microorganismal homeostasis. If the decision to review a research proposal is based on specific genetic elements instead of the taxa, then fewer proposals would need to be reviewed in a new oversight regime, and the review process would involve checking if the sequences of the proposal in question are found on the list.

In a new SoC regulatory regime, researchers and regulators from approved and registered institutions should be able to search an officially endorsed and maintained dataset of SoCs by (1) name/taxa of a SoC, (2) accession number (UniProt or NCBI, possibly gene number), and (3) nucleotide/protein sequence. However, access to the dataset of SoCs should be limited. Registration of institutions and an approval process that ensures proper identification should be necessary to view an entry. The full version is an information hazard, so it should not be accessible anonymously or downloaded, but it must be queryable in at least the ways mentioned above. How secure access to such datasets might be managed has been discussed by others.^3,39^

There is a worry that depositing annotated SoCs into public datasets as we propose to do could enable them to be employed for vile purposes. Even though all of these sequences have been described in the publicly available literature, often for several decades, aggregating them under such a set of terms puts them a step closer to recognition. While true, this situation has already existed for viruses since 2015.^40^ Also, understanding the biothreat implications of a sequence from the perspective of biological engineering is not as simple as merely aggregating the terms. There is a considerable tacit knowledge required, some of which would need to be instantiated in any “official” dataset of SoCs. A government-approved set of SoCs, used for screening synthetic sequences, might require more detailed descriptions of the sequences with respect to how they might be misused. There could also be a scoring or ranking system for which ones were more problematic. In our experience, such a system would necessarily be subjective, but it could be made internally consistent. In any case, we think the benefits of publicly available, improved annotations of SoCs for infectious disease research outweighs the risks.

## Drawing a Line: Pathogens of Which Hosts?

While SoCs need to be obtained and categorized so as to properly assess DURC, not every pathogen needs to have its sequences, even the ones necessary for pathogenesis, so treated. What should not be systematically gathered from the literature are sequences from pathogens of hosts that do not have implications for national security. Similarly, the impacts of certain pathogens on national security may be so small as to not be worth regulating. The recently issued executive order explicitly seeks “to establish criteria and mechanisms for ongoing identification of biological sequences that could be used in a manner that would pose a risk to the national security of the United States.”^4^ The level of risk to be accepted is a judgment call. Prioritization with regard to hosts is advisable; prudential considerations are appropriate. Defending human aquaculture by documenting SoCs from pathogens that only affect mollusks, crustaceans, and fish may not be worthwhile. One justifiable measure for choosing whether to find and annotate SoCs from pathogens from particular hosts is how much those hosts contribute to the well-being of the citizens of the United States. SoCs from all human pathogens, whether immunocompetent or immunodeficient (opportunistic pathogens) should be documented.

## Conclusion

We have been working to improve the controlled vocabularies required for an adequate description of multiorganism interactions, particularly pathogenic interactions resulting in deleterious consequences for the host (or target). We have previously used provisional terms to categorize thousands of pathogen sequences that mediate these interactions. The databases we discussed are all committed to presenting important molecular information on pathogenic microbes in a convenient and accessible format. However, they cannot sufficiently describe biothreats since they lack a standardized description of SoCs involved in microbial pathogenesis in host organisms. In addition, they suffer, as do all datasets, from an inability to annotate sequences, as fast as their investigations are published, to the exacting requirements desired by any and all conceivable users.

The set of GO terms we are developing will be apt for describing the pathogenic interactions regularly employed by pathogen SoCs to overcome host barriers, subvert innate immune systems, disseminate within hosts, and otherwise negatively affect critical components of host organisms. This set of terms will be readily adaptable to describing sequences collected for screening synthetic DNA and for assessing biothreats. Better annotation of these sequences is needed as the United States moves to a biosecurity regulatory framework that considers more than simply the taxonomy of problematic microbes and also how the sequences mediating pathogenesis contribute to that state.
